# Long-Term Operational Stability of Ta/Pt Thin-Film Microheaters: Impact of the Ta Adhesion Layer

**DOI:** 10.3390/nano13010094

**Published:** 2022-12-25

**Authors:** Ivan A. Kalinin, Ilya V. Roslyakov, Dmitry N. Khmelenin, Kirill S. Napolskii

**Affiliations:** 1 Department of Materials Science, Lomonosov Moscow State University, 119991 Moscow, Russia; 2 Department of Chemistry, Lomonosov Moscow State University, 119991 Moscow, Russia; 3 Shubnikov Institute of Crystallography of Federal Scientific Research Center ‘Crystallography and Photonics’, Russian Academy of Sciences, 119333 Moscow, Russia

**Keywords:** microheater, adhesion layer, anodic aluminium oxide, thermal stability, resistance drift

## Abstract

Microheaters with long-term stability are crucial for the development of a variety of microelectronic devices operated at high temperatures. Structured Ta/Pt bilayers, in which the Ta sublayer ensures high adhesion of the Pt resistive layer, are widely used to create microheaters. Herein, a comprehensive study of the microstructure of Ta/Pt films using high-resolution transmission electron microscopy with local elemental analysis reveals the twofold nature of Ta after annealing. The main fraction of Ta persists in the form of tantalum oxide between the Pt resistive layer and the alumina substrate. Such a sublayer hampers Pt recrystallization and grain growth in bilayered Ta/Pt films in comparison with pure Pt films. Tantalum is also observed inside the Pt grains as individual Ta nanoparticles, but their volume fraction is only about 2%. Microheaters based on the 10 nm Ta/90 nm Pt bilayers after pre-annealing exhibit long-term stability with low resistance drift at 500 °C (less than 3%/month).

## 1. Introduction

Resistive (Joule) heating elements are widely used in science and technology. The current trend to thin-film microheaters (microhotplates) has numerous advantages, such as compact device size, low power consumption, fast response time, and improved reproducibility, owing to the application of well-automated microelectronic fabrication techniques. Thin-film microheaters are used as an integral part of semiconductor [1,2] and thermocatalytic [3,4,5] gas sensors, gas flow rate sensors [6,7], and fuel cells [8]; they can be applied to high-temperature in situ microscopy [9,10,11], microfluidic chips [12], quartz crystal microbalances [13], micrometer-scale phase modulators [14] and thermoelectrics [15,16].

Typically, the resistive layer of a microheater is made of metals [17,18,19,20], titanium nitride (TiN) [21,22] or polysilicon [23,24]. Among these materials, platinum (Pt) is the most commonly used one. Pt possesses a high chemical inertness and a high melting point (1768 °C), necessary for stable operation at high temperatures, and a high and constant temperature coefficient of resistance (TCR) (3.9 × 10^3^ ppm/°C [25]) over a wide temperature range, which is essential for the accurate control of the microheater temperature.

The direct deposition of Pt on oxide or nitride substrates results in the low stability of such systems at high temperatures [26,27] due to poor adhesion leading to the delamination of the metal from the substrate [27,28]. The chemical interaction of Pt with silicon-based substrates [29,30] is also possible. A thin adhesion layer with a high affinity for oxygen allows one to solve these problems being introduced between the resistive layer and the substrate. Usually, high-reactive metals that form strong (chemical) bonds with a non-conductive substrate and metal-metal bonds with Pt or its alloys serve as effective adhesion layers. In particular, titanium (Ti) is often used for this purpose [12,31,32,33,34,35,36,37,38] despite some limitations, e.g., the mutual diffusion between Ti and Pt [39,40,41,42] and the formation of a Pt_3_Ti intermetallic compound [43,44,45]. As a result, significant change in the operating parameters makes long-term operation of microheaters based on Ti/Pt bilayers impossible. Similar problems are observed for chromium (Cr) [46,47,48], aluminum (Al) [47], zirconium (Zr) [47,49,50,51,52], and hafnium (Hf) [47,49] adhesion layer materials. In the case of tantalum (Ta) adhesion layer [53,54,55,56], intermetallic compounds are not observed. However, studies of the mutual diffusion between Ta and Pt layers and its effect on the operation parameters of microheaters are scarce [49,57,58,59,60].

Thus, the present work focuses on investigating the electrical properties, morphology, and composition of Ta/Pt bilayer thin films after annealing. Understanding the processes occurring at the substrate/adhesion layer (Ta)/resistive layer (Pt) interfaces is crucial for developing reliable, long-life devices. Porous anodic aluminium oxide (AAO), which is promising in place of the more widespread substrates based on silicon compounds [4,61], is chosen as a substrate to create microheaters.

## 2. Materials and Methods

To obtain thin-film microheaters, the experimental approach described in detail in our previous work [62] was used. Porous AAO was obtained by anodization of aluminium in 0.3 M oxalic acid at 120 V and an electrolyte temperature of 1 ± 1 °C. Before anodization, the aluminium foil (0.5 mm thick, 99.999% purity) was mechanically and electrochemically polished [63] to a mirror finish. During anodization, the voltage was swept with a rate of 0.5 V/s up to 120 V and then kept constant until the electric charge density reached 60 C/cm^2^. The thickness of AAO under these conditions equals 30 μm. On the upper surface of the AAO substrate, the pore diameter is 32 ± 15 nm (Figure S1).

An array of thin-film microheaters was prepared by lift-off photolithography of DC magnetron sputtered metal layers. A 90 nm thick Pt film and bilayer 10 nm Ta/90 nm Pt thin films were sputtered onto the AAO substrate using a Quorum Technologies Q300T D Plus magnetron deposition system (Lewes, UK). A residual pressure of 10^−4^ mbar and working Ar pressure of 10^−2^ mbar were used. Ta and Pt layers were sputtered consecutively from the two individual targets without vacuum breaking. Thereafter, the array of microheaters was separated into individual microchips with an area of 2×2 mm^2^ by chemical photolithography. A solution of 0.6 M H_3_PO_4_ at 60 °C was used as an etchant of AAO substrate. Further, the underlying aluminium foil was selectively dissolved in 0.25 M CuCl_2_ and 0.68 M HCl solution.

**Figure 1:**
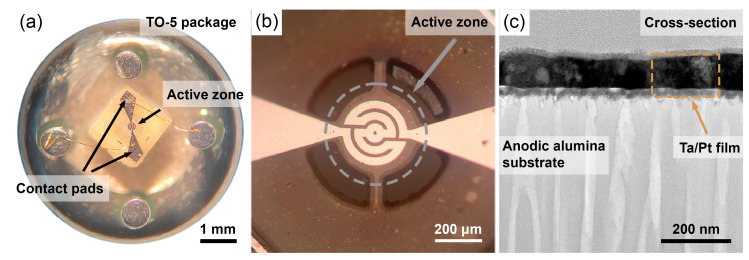
Microheater mounted in TO-5 package (**a**). Optical image of the active zone of the microheater (**b**). Cross-section of a Ta/Pt thin film on a porous anodic aluminium oxide substrate (**c**).

Free-standing AAO chips with microheaters were annealed at 600, 730, and 810 °C (heating rate of 2 °C/min) for 12 h in a muffle furnace Nabertherm L5/12 (Lilienthal, Germany) under a 4 g/cm^2^ load to prevent bending of the substrate. Finally, the microchips were mounted in TO-5 packages [64] (Figure 1a). The contact pads of the microheaters were connected to the package pins by a Kulicke and Soffa Model 4526 (Singapore) wedge wire bonder using a 25 µm thick Au wire.

Transmission electron microscopy (TEM) of the samples was realized using a Tecnai Osiris FEI microscope (Hillsboro, OR, USA). A focused ion beam (FIB) was used to cut the cross-section lamella of specimens for TEM investigation. Scanning electron microscopy (SEM) images of the microheaters were collected using a Leo Supra 50 VP instrument (Oberkochen, Germany).

The active zone of a microheater consists of a two-dimensional metal spiral (Figure 1b). The spiral track width is 32 µm, and the distance between the tracks is 16 µm. The total length of the spiral is about 1 mm. TEM measurements of the cross-section of a bilayer thin film reveal a thickness of 95 ± 9 nm of the bilayer Ta/Pt film (Figure 1c).

## 3. Results and Discussion

**Figure 2:**
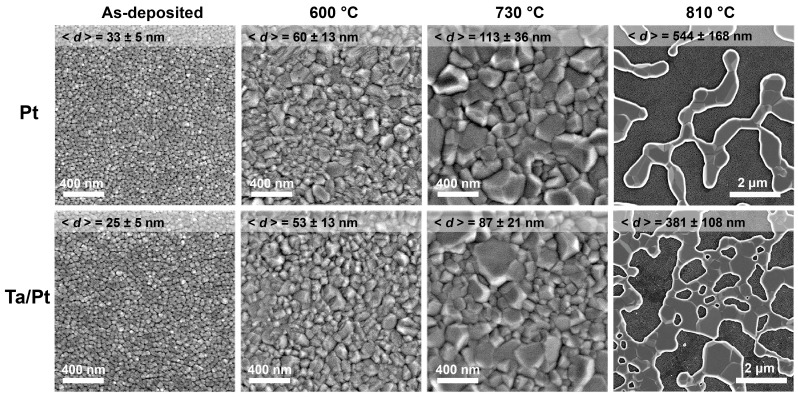
Scanning electron microscopy images of the surface of thin-film microheaters after annealing at different temperatures for 12 h at a heating rate of 2 °C/min. The average grain size is indicated at the top of each image.

The morphology of as-deposited Ta/Pt films and Ta/Pt films annealed at 600 and 730 °C is similar to that of the corresponding Pt films (Figure 2). However, statistical analysis of the SEM images using ImageJ software [65] elucidated a smaller average grain size (<*d*>) for microheaters with a Ta adhesion layer in comparison with pure Pt. Recrystallization at 810 °C led to significant degradation of the Pt film and the formation of a non-conductive structure consisting of the separate metal islands. After annealing under the same conditions, the Ta/Pt film retained integrity despite the formation of many voids. Smaller <*d*> values and a smaller size of the defect areas in the case of thin films with the Ta adhesion layer was due to a decrease in the diffusion mobility of Pt, indicating greater high-temperature stability of the bilayer microheaters.

Table 1 shows the electrical characteristics of the Pt and Ta/Pt microheaters depending on the annealing temperature. The TCR of the microheaters was obtained by linearization of the temperature dependence of resistance. For this purpose, the resistance measurements were performed in a Binder FD 23 heating chamber (Tuttlingen, Germany) in the temperature range from 30 to 90 °C with a step of 20 °C (Figure S2). The dependencies of the current, temperature, and power consumption of the microheaters on the applied voltage were obtained from cyclic voltammograms (Figure S3). A linear voltage sweep with a rate of 250 mV/s was performed using an Autolab PGSTAT302N (Herisau, Switzerland) in the range of 0–6 V. The long-term resistance drift was measured at a constant supply voltage corresponding to the active zone temperature of 500 ± 20 °C after ageing at this temperature for 20 h.

The recrystallization of thin films at temperatures of 600 and 730 °C led to a resistance decrease due to the growth of Pt grains and, as a consequence, a reduction of electron scattering at the grain boundaries. The resistance of microheaters increased after annealing at 810 °C due to the formation of large-scale voids (Figure 2, right column). In the case of Pt microheaters without the Ta adhesion layer, a complete loss of electrical conductivity was observed after annealing at 810 °C. The TCR of thin-film microheaters rose with the grain growth to about 3 × 10^3^ ppm/°C. The supply voltage and power consumption of microheaters are given for the active zone temperature of 500 °C, which is a typical operating temperature of catalytic and semiconductor gas sensors [3,66]. These values are almost independent of the composition of microheaters and annealing conditions. The microheaters without an adhesion layer lost conductivity due to continuous recrystallization at the ageing stage lasting 20 h. Therefore, it was impossible to study the long-term resistance drift for a longer period of time. Among the Ta/Pt microheaters, thin films annealed at 730 °C for 12 h demonstrate the best performance: the resistance drift during continuous operation at the active zone temperature of 500 °C is about 3% per month.

**Figure 3:**
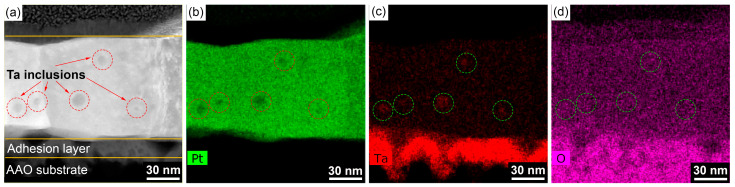
High-angle annular dark-field scanning transmission electron microscopy of the cross-section of Ta/Pt film annealed at 730 °C for 12 h (**a**). Energy-dispersive X-ray spectroscopy maps of the elements Pt (**b**), Ta (**c**), and O (**d**).

From a morphological point of view, the Ta/Pt layer annealed at 730 °C for 12 h consists of large uniform crystallites without voids and abnormally large grains. The cross-section structure of this sample was studied in detail using TEM-based techniques. The STEM image (Figure 3a) reveals a columnar structure of the Pt resistive layer, which consists of grains with height equal to the film thickness. Such a morphology is typical for Pt films obtained by magnetron sputtering [45,67,68]. Several round-shaped particles are included inside the grains and are mainly located near the AAO substrate. According to the Z-contrast, the observed particles possess a lower average atomic number compared to Pt. Their volume fraction in the Pt matrix is about 2 vol.%. The EDS maps (Figure 3b-d) indicate the Ta-enrichment of these particles compared to the bulk of the Pt film. In the area of inclusions, the Ta map does not overlap with the O map, manifesting that the particles consist of metallic Ta. Any segregation of Ta along the grain boundaries was not observed. Thus, we can conclude that the effect of the adhesion layer on the electrical properties of the Pt film should be negligible.

**Figure 4:**
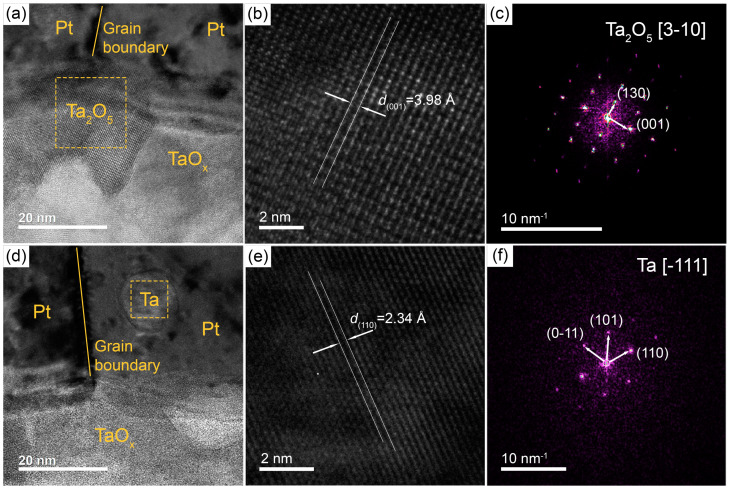
Cross-sectional high-resolution transmission electron microscopy (HRTEM) images of the Ta/Pt film annealed at 730 °C for 12 h (**a**,**d**) with zoomed images showing lattice structure of boxed area (**b**,**e**) and corresponding FFT patterns (**c**,**f**).

The overlapping of the Ta and O maps in the area of the adhesion layer suggests the Ta oxidation between Pt resistive layer and AAO substrate. An enlarged image of the Ta adhesion layer (Figure 4a) shows crystallites with an average size of about 20 nm. FFT analysis of the selected area of the high-resolution TEM (HRTEM) image (Figure 4b,c) performed by Gwyddion software [69] allows us to recognize the Ta_2_O_5_ phase (ICDD PDF-2 71-639). Thus, after annealing at 730 °C for 12 h in air, the Ta adhesion layer is almost completely oxidized.

The Ta inclusions inside the Pt grains are visualized well in the HRTEM images (Figure 4d). According to the FFT analysis of inclusions (Figure 4e,f), the interplanar spacing and angles between the lattice vectors correspond to the phase of metallic Ta (ICDD PDF-2 4-788). Thus, recrystallization of Ta/Pt films leads to the localization of metallic Ta particles with a deformed crystal lattice inside the Pt grains.

Thus, we found twofold nature of tantalum in Ta/Pt thin-film microheaters. The main fraction of Ta is located between the Pt resistive layer and the AAO substrate in the form of crystalline tantalum oxide, consistent with earlier results [49,58,60,70]. Oxygen diffusion to the Ta layer may occur both through the Pt film by the mechanism of grain boundary migration [39] and through the porous AAO substrate. There is no significant diffusion of the Ta adhesion layer into the Pt resistive layer during recrystallization. It agrees well with the data reported in Refs. [58,60,71], where bulk analysis of the compositional depth profiles using Auger electron spectroscopy and Rutherford backscattering spectrometry were performed.

Additionally, local TEM analysis reveals a small amount of metallic Ta particles, which are localized mainly in the lower part of the Pt grains after recrystallization. Their volume fraction is about 2%, and, consequently, the influence of Ta nanoparticles on the characteristics of the Pt resistive layer is insignificant. It should be noted that the EDS maps confirm the absence of such particles in the as-deposited Ta/Pt film before recrystallization (Figure S4). As a probable mechanism of Ta particle incorporation inside Pt grains, we can assume the following: during magnetron sputtering, high-energy atoms and atomic clusters of Pt get onto the Ta adhesion layer with the formation of a thin mixed layer between Ta and Pt atoms and/or atomic clusters. Thus, a certain amount of uniformly distributed Ta atoms appears in the lower part of the Pt film. Subsequent recrystallization of Pt leads to the agglomeration of Ta atoms to nanoparticles inside Pt grains.

## 4. Conclusions

In summary, a comprehensive study of the influence of the Ta adhesion layer on the microstructure of Pt films and the electrical properties of microheaters was carried out. The preliminary recrystallization of the bilayered structure, based on the 10 nm Ta adhesion layer and 90 nm Pt resistive layer, at 730 °C for 12 h allows one to obtain microheaters with long-term operational stability. Resistance drift during operation at 500 °C is less than 3%/month. The Ta adhesion layer after annealing consists of the 20 nm sized Ta_2_O_5_ crystallites. Additionally, individual particles of metallic Ta with an average size of 15 nm were observed inside the 100 nm grains of the resistive Pt layer. These particles are mainly located near the adhesion layer, and their volume fraction is about 2 vol.%.

## Figures and Tables

**Table 1:** Parameters of Pt and Ta/Pt microheaters annealed at different temperatures for 12 h. The data was averaged over at least three microheaters in each case. The duration of the resistance drift measurement is 1 week.

Characteristic	As-Deposited		600 °C		730 °C		810 °C	
	Pt	Ta/Pt	Pt	Ta/Pt	Pt	Ta/Pt	Pt	Ta/Pt
Resistance, Ω	131 ± 1	86 ± 4	73 ± 3	66 ± 1	68 ± 5	69 ± 4	-	97 ± 10
TCR (×10^3^), ppm/°C	1.5 ± 0.1	2.0 ± 0.1	2.8 ± 0.1	3.1 ± 0.1	3.1 ± 0.3	3.1 ± 0.2	-	3.3 ± 0.3
Power consumption at 500 °C, mW	-	-	101 ± 3	120 ± 17	99 ± 5	113 ± 10	-	101 ± 8
Supply voltage (500 °C), V	-	-	4.3 ± 0.1	4.4 ± 0.3	4.0 ± 0.1	4.4 ± 0.3	-	5.1 ± 0.2
Resistance drift, %/day	-	-	-	0.19 ± 0.14	-	0.09 ± 0.03	-	0.17 ± 0.07

## Data Availability

Not applicable.

## Supplementary Materials

The following supporting information can be downloaded at: https://www.mdpi.com/article/10.3390/nano13010094/s1, Figure S1: SEM image of the AAO substrate before metal sputtering, Figure S2: Determination of TCR of microheaters; Figure S3: Dependences of the active zone temperature and power consumption of microheaters on the supply voltage; Figure S4: TEM image of the as-deposited Ta/Pt film and EDS maps of Pt and Ta.
