# Supplementary material for: Long-Term Operational Stability of Ta/Pt Thin-Film Microheaters: Impact of the Ta Adhesion Layer
Source: Nanomaterials (Basel). 2022 Dec 25;13(1):94. doi: 10.3390/nano13010094 (PMC9824110; doi:10.3390/nano13010094)
Supplement: Supplementary file 1 [file nanomaterials-13-00094-s001.zip › nanomaterials-2083266-supplementary.pdf]

Supplementary materials

Long-term operational stability of Ta/Pt thin film microheaters: impact of the Ta adhesion layer

Ivan A. Kalinin [1,2,*], Ilya V. Roslyakov [1], Dmitry N. Khmelenin [3] and Kirill S. Napolskii [1,2,*]

[1] Department of Materials Science, Lomonosov Moscow State University, 119991, Moscow, Russia
[2] Department of Chemistry, Lomonosov Moscow State University, 119991, Moscow, Russia
[3] Shubnikov Institute of Crystallography of Federal Scientific Research Center 'Crystallography and Photonics', Russian Academy of Sciences, 119333, Moscow, Russia
* Correspondence: ikalinin@inorg.chem.msu.ru (I.A.K.); kirill@inorg.chem.msu.ru (K.S.N.)

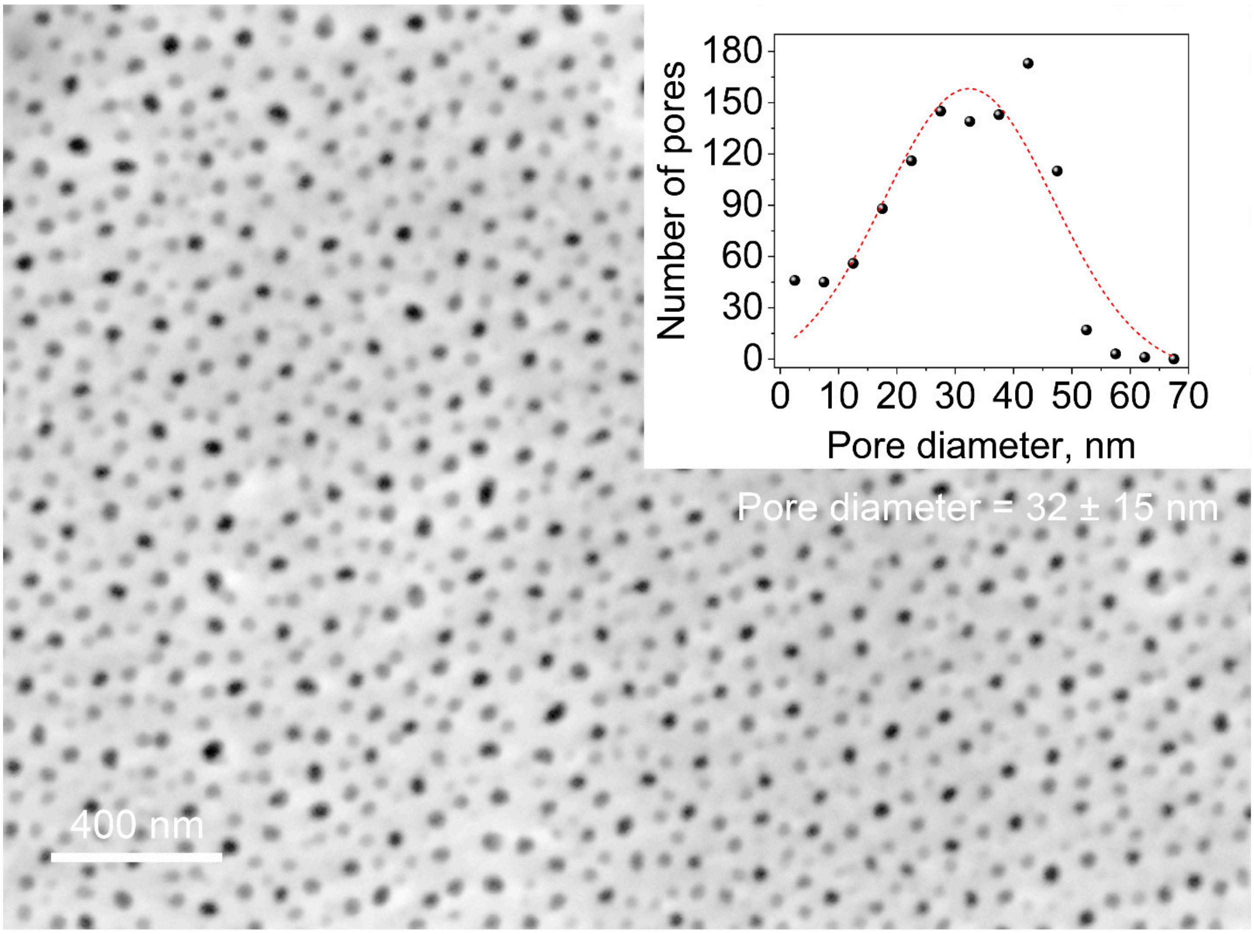

Figure S1. The SEM image of the porous AAO film obtained at 120 V in 0.3 M oxalic acid. The inset shows pore size distribution.

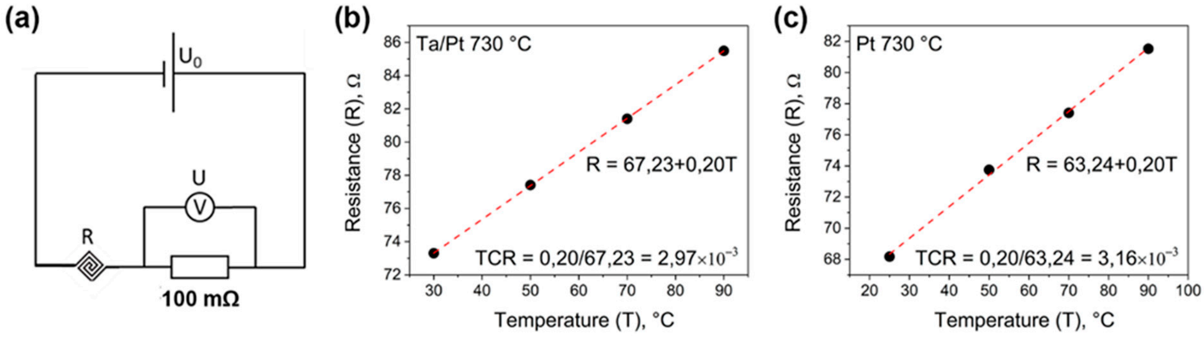

Figure S2. The electrical circuit used to determine the TCR of microheaters (a) and the temperature dependences of the electrical resistance of Ta/Pt (b) and Pt (c) microheaters pre-annealed at 730 °C.

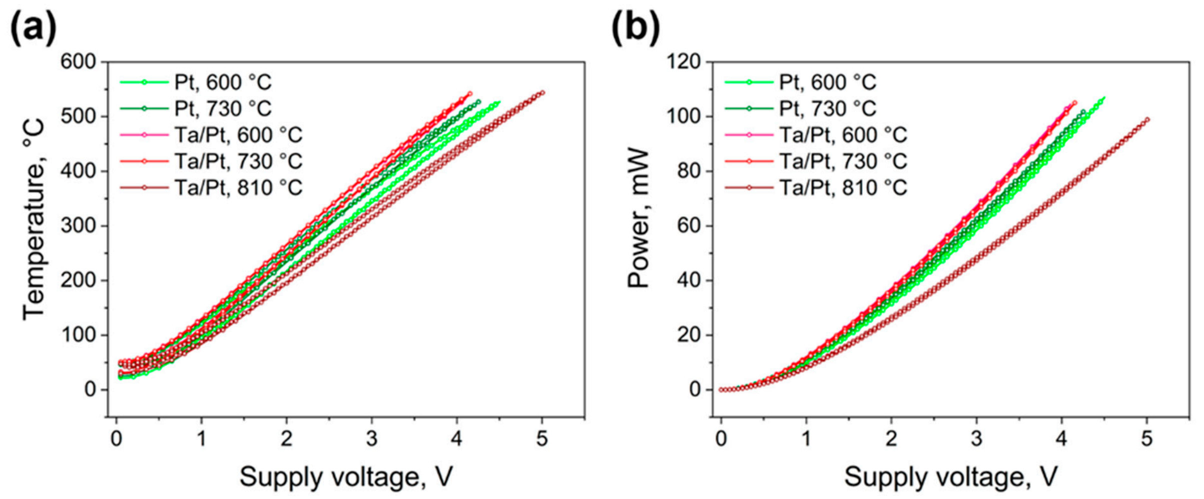

Figure S3. Determination of the electrical characteristics of microheaters. Examples of the dependences of the active zone temperature (a) and power consumption (b) on the supply voltage. The composition of microheaters and the pre-annealing temperature are shown in the legends.

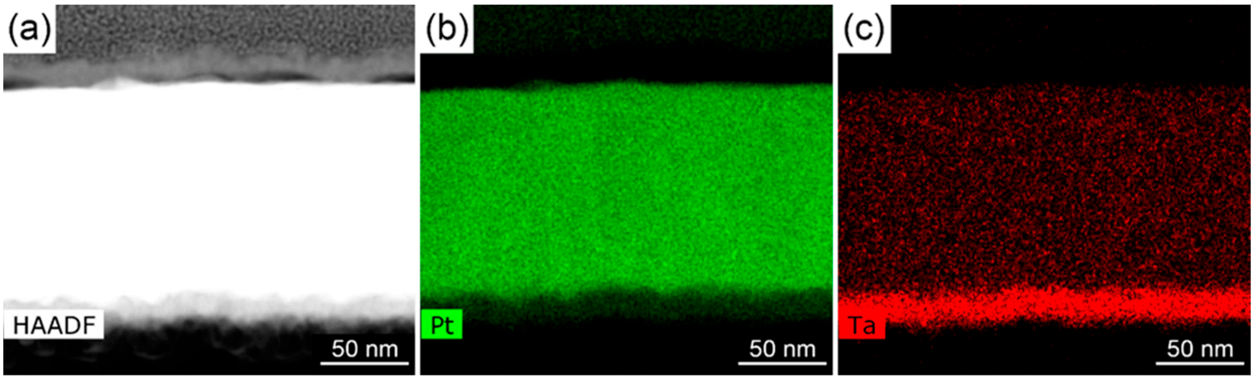

Figure S4. High-angle annular dark-field scanning transmission electron microscopy of as-deposited Ta/Pt film (a). Energy-dispersive X-ray spectroscopy maps of the elements: Pt (b) and Ta (c).
